# Real-world effect of the treatment for painful subacute thyroiditis: The combined injection of lidocaine and triamcinolone using an insulin pen

**DOI:** 10.20945/2359-3997000000542

**Published:** 2022-12-01

**Authors:** Xu Hu, Hairong Hao, Manli Guo, Shao-gang Ma

**Affiliations:** 1 Huai'an Hospital Affiliated to Xuzhou Medical College and Huai'an Second People's Hospital Department of Endocrinology and Metabolism Huai'an China Department of Endocrinology and Metabolism, Huai'an Hospital Affiliated to Xuzhou Medical College and Huai'an Second People's Hospital, Huai'an 223002, China; 2 The Affiliated Suqian Hospital of Xuzhou Medical University Nanjing Drum Tower Hospital Group Suqian Hospital Department of Endocrinology and Metabolism Suqian China Department of Endocrinology and Metabolism, The Affiliated Suqian Hospital of Xuzhou Medical University, and Nanjing Drum Tower Hospital Group Suqian Hospital, Suqian 223800, China; 3 Laibin People's Hospital Department of Endocrinology and Metabolism Laibin China Department of Endocrinology and Metabolism, Laibin People's Hospital, Laibin 546100, China

**Keywords:** Subacute thyroiditis, intrathyroidal injection, evaluation

## Abstract

**Objective:**

Intrathyroidal injection using an insulin pen filled with a mixture of lidocaine and triamcinolone acetonide is a therapy for subacute thyroiditis (SAT) reported by us previously. We aimed to evaluate the clinical efficacy of ultrasound-guided intrathyroidal injection in the treatment of SAT.

**Subjects and methods:**

A total of 93 patients with SAT completed the study. All patients were evaluated via a history and clinical examination followed by thyroid function tests and ultrasonography of the thyroid. After ultrasound-guided intrathyroidal injection, the patients were followed up with respect to the injection frequency, treatment duration, and patient satisfaction. The visual numerical rating scale was used as a pain questionnaire for a given interval.

**Results:**

Thyroid pain instantly decreased to scores below 3.0 following the first injection. Sixty-three patients (67.74%) avoided relapse of thyroid pain within 3 injections, which occurred within only 3 days after the first injection. The pain in 27 patients (29.03%) disappeared completely after 4-6 injections. Only 3 patients (3.23%) were found to need more than 6 injections, with 10 cited as the maximum number of injections, the injection took only 17 days altogether. The mean treatment cycle of the intrathyroidal injection was 3.98 days. There were no other associated complications with the novel therapy except infrequent small subcutaneous hematomas, which could be prevented with skilled practice. The average patient satisfaction score was as high as 9.0.

**Conclusion:**

Intrathyroidal injection of lidocaine and triamcinolone acetonide using an insulin pen was found to be an advantageous and satisfactory treatment for SAT.

## INTRODUCTION

Subacute thyroiditis (SAT), a transient and self-limiting inflammatory thyroid disease, has gradually increased, and its etiology remains unclear. According to statistical analysis, the incidence of SAT is 4.9/100,000 persons, accounting for 5% of all thyroid diseases (1). SAT often occurs in 30- to 50-year-old females. In addition, 22.8% to 26.8% of patients develop permanent hypothyroidism (2,3). The distinctive manifestation of SAT is mainly severe pain, which may be limited to the thyroid region and radiate from the neck to the jaw, throat, upper chest or ears. Other clinical presentations included fever, fatigue, thyroid tenderness and enlargement. At the early stage, the laboratory examination shows thyrotoxicosis, a decreased thyroid iodine uptake rate, and elevated erythrocyte sedimentation rate and C-reactive protein levels (4,5). Ultrasonography often demonstrates a heterogeneous low-echo region with fuzzy boundaries in the thyroid gland, accompanied by abundant blood flow signals in the periphery with fewer signals in the internal region (6).

The initial use of nonsteroidal anti-inflammatory drugs (NSAIDs) was recommend, particularly for mild SAT cases. Oral glucocorticoids (GCs) are suggested when patients fail to respond to treatment or initially present with moderate to severe pain and/or thyrotoxic symptoms (7,8). Symptomatic relief is achieved with these drugs, but these therapies result in slow effects, a delayed course of illness and many side effects. There were no definitive recommendations on the medical management of SAT (9). Moreover, significant attention should be given to the side effects of GCs therapy, including peptic ulcers, increased appetite, and adrenocortical dysfunction. The administration of oral glucocorticoids in SAT patients for an extended period of time will cause metabolic changes. Hence, it is necessary to develop a new effective therapeutic strategy for SAT.

In our previous report, a total of 18 patients who underwent intrathyroidal injection reported rapid pain relief and significant neck relaxation within 1 week compared with the oral prednisone group (10). The frequency and duration of treatments were significantly lower in these patients. However, the study had small sample sizes, which limited its value as a clinical reference. At present, we aimed to re-evaluate the clinical efficacy and safety of ultrasound-guided intrathyroidal injection in the treatment of SAT in a larger sample.

## SUBJECTS AND METHODS

### Patients

A total of 126 Chinese patients with newly diagnosed painful SAT (all with acute anterior neck pain) who underwent treatment at Huai'an Second People's Hospital from June 2014 to July 2019 were included in this study. The diagnosis of SAT was based on laboratory findings that revealed elevated ESR (>20 mm/hour) or at least C-reactive protein (>10 mg/L) and hypoechoic areas/areas with blurred margins and decreased vascularization on ultrasound.

Meanwhile, at least one of the following criteria should be met: 1. Hard and swollen thyroid. 2. Pain and tenderness of the thyroid gland/lobe. 3. Elevated serum FT4 and suppressed TSH. 4. Decreased radioiodine uptake. 5. Typical FNAB result for SAT (11). All 126 SAT patients with acute anterior neck pain for less than or equal to three days were included in the study.

The study was approved by the ethics committee of Huai'an Second People's Hospital, and the code of approval is No. 20141209. Informed consent was obtained from all of the participants who were informed about the study, procedure, outcomes and complications. The exclusion criteria were as follows: pregnancy, severe heart disease, end-stage renal and liver diseases and a history of allergy to lidocaine and glucocorticoids.

### Study design

#### Operation process of injection

At study entry, all participants received a clinical evaluation for the collection of experimental data. The treatment adopted in this study was intrathyroid injection with an insulin pen under ultrasound guidance. Due to expansion and compression of the thyroid parenchyma swelling toward the skin, the distance from the skin to the thyroid capsule was shortened to 2-3 mm in the isthmus and 3-5 mm in the lateral lobes of SAT patients. Based on the distance from the skin to the thyroid capsule in SAT patients, the thyroid isthmus was injected with a 4-mm needle (32 G), and the lobe was injected with a 6-8 mm needle (32 or 30 G). The thyroid was scanned via ultrasound before the operation to understand the location, boundary, morphology, and internal echo of the pathological tissue in both lobes. The injection was administered in the pathological tissue of the lobe of the thyroid (low-echo region). The injection needle was perpendicular to the skin at the angle of 90°. Target the low echo area (the pathological tissue) for injection and pause for at least 5 seconds after pressing the injection button. After the injection, the injection sites needs to be pressed for about 10 seconds. If bleeding occurs, press a sterile cotton ball on the skin for about 30 seconds or longer.

#### Recycling of syringes

The insulin penfills were rinsed and recycled after the insulin was injected. The insulin refill that was used was removed (the volume was generally 3 mL), and approximately 3 mL of sterile normal saline was injected into it with a 5 mL syringe. Drain and inject the sterile normal saline repeatedly (five to ten times). To achieve the effect of repeated washing, the syringe needle would not be pulled out, except when replacing new sterile normal saline three times.

#### Drug dose of injection

The penfills were filled with 3.0 mL (300 units) solution that contained 2 mL lidocaine solution (drug concentration 5 mL: 0.1 g), 1 mL triamcinolone acetonide solution (drug concentration 5 mL:50 mg). The doses of lidocaine and triamcinolone were 40 mg and 10 mg, respectively. The painful lobe was injected with 20 to 80 Units (calibration of insulin pen), which was divided into five locations of the inflammatory site (low-echo region in the ultrasound): up, down, left and right, and the center. The initial dose of injected drugs depended on the size of the low echo region. It required 40 units of the injection drugs when the region of low echo area is 1 to 1.5 cm in diameter. It required 60 units of the injection drugs when the region of low echo area is 1.5 to 2 cm in diameter. It required 80 units of the injection drugs when the region of low echo area is more than 2 cm in diameter.

#### Duration of injection

A total of 3 injections were administered daily for the first three days and then every other day, wherein the injections were given for at least three days until the patients were satisfied with the level of pain relief. After symptom relief or disappearance for more than one day, ultrasound examination was performed. Therapy was discontinued as soon as the thyroid morphology returned to normal. If it recurred anytime during hospitalization, the treatment was continued. Thyroid function, routine blood count, and the ESR were reviewed to observe the safety and efficacy of the treatment. At the end of the first week, if the patients receiving injections continued to complain of significant pain in the thyroid region after five injections, oral ibuprofen was added to the regimen. At the beginning of the third week, if SAT persisted or recurred, prednisone or ibuprofen was administered orally.

#### Therapeutic evaluation

By verbally administered numerical rating scales (NRSs), patients verbally rated pain intensity as an integer from 0 to 10 (0 = no pain, 10 = worst possible pain) and marked their pain intensity on a 10-cm horizontal visual analog scale (VAS) bounded by these descriptors (12). The thyroid pain score (0-10) was assessed at presentation, 30 seconds and 60 seconds after treatment, 30 and 60 minutes after treatment, and 24 hours after treatment. Patients were asked whether their pain was “much less”, “slightly less”, “about the same”, “slightly more”, or “much more” after the treatment (“If your pain score was 10 at presentation, what is it now?”).

Verbally administered numerical rating scales were also used to measure satisfaction with the therapy. Patients verbally graded satisfaction as an integer from 0 to 10 (0 = not at all satisfied, 10 = very satisfied) and marked their satisfaction on a 10-cm horizontal visual analog scale (VAS) bounded by these descriptors.

The clinical features and laboratory measurements before and after treatment were recorded and compared, such as body temperature, swelling with pain (using numerical rating scales), tenderness in the thyroid gland, neck pain, thyroid function, routine blood count, and ESR and CRP levels. The duration of the treatment, the injection times and the patient satisfaction were observed and analyzed.

Evaluation of clinical efficacy: 1. Recovery: the body temperature returned to normal, the symptoms of neck pain disappeared (VAS < 3’), and the goiter returned to normal expected characteristics. ESR decreased to below 20 mm/h, and thyroid function returned to normal. 2. Effective: body temperature returned to normal, neck pain significantly improved, thyroid enlargement was smaller than before but not completely normal, ESR was close to normal (20-25 mm/h) when any of the above conditions were met, and thyroid function was improved. 3. Ineffective: fever, symptoms of neck pain were still evident (VAS > 4 points), ESR was still very fast, the goiter did not change, and thyroid function was higher than normal, in accordance with any of the above. 4. Worsening: fever, aggravated thyroid pain (VAS > 7 points), the sense of distension was not relieved, ESR was faster than before, and thyroid function was considered hyperactive.

#### Adverse reactions and complications

The following adverse reactions and complications were evaluated whether to occur: patients were observed whether to have pain and local congestion caused by percutaneous intrathyroid injection or whether to have symptoms of nerve injury, such as hoarseness, a choking cough when drinking water, abnormal breathing, tracheal perforation, or serious adverse events such as syncope, shock or loss of consciousness.

#### Follow-up

After the therapy was discontinued, follow-up visits were performed every 1 month until half a year later. In addition, patients were reviewed immediately if they felt they might be suffering from disease relapse.

#### Clinical assays

Blood samples were drawn from the subjects by venipuncture into vacutainer tubes. The serum levels of TSH, FT3, FT4, and anti-thyroid antibodies were determined by electrochemiluminescence immunoassays (The Roche Cobas 6000, Roche Diagnostics, Indianapolis, IN). The reference ranges were as follows: TSH 0.34-5.44 mIU/mL, FT3 2.92-5.93 pmol/L, FT4 7.91-20.59 pmol/L, WBCs (4.0-10.0) × 10^9^/L, neutrophils (1.80-6.30)×10^9^/L, ESR 0-20.0 mm/h, and CRP 0-5.0 mg/L. In addition, the distance between the skin surface and the thyroid capsule for all the patients was obtained by thyroid ultrasound. The anti-TSH receptor antibody (TSHR-Ab) titer and I-131 uptake was measured to confirm the diagnosis. Laboratory tests, including the white blood cell count, the ESR, and other routine tests, were obtained by routine clinical assays in the hospital laboratory.

#### Statistical methods

Statistical analyses were conducted with the SPSS 18.0 software (SPSS Inc., Chicago, IL). The normally distributed measurement data are represented as the mean ± standard deviation. The skewed measurement data are expressed as the median (minimum - maximum). And count data are expressed as a percentage (%). The Student's paired t test was used to compare the mean values before and after treatment. A two-tailed *p* value < 0.05 was considered statistically significant.

## RESULTS

### Clinical and biochemical characteristics

The clinical and biochemical characteristics of the SAT patients are presented in Table 1. Of 126 patients with SAT who were screened, 33 patients did not complete the follow-up. Therefore, a final analysis was performed on the 93 patients who underwent regular follow-up. The ages of the patients ranged from 20 years to 82 years, with a mean age of 46.83 years. Females accounted for 65 patients (69.9%), and males accounted for 28 patients (30.1%). The female to male ratio was 2.32:1. All patients reported severe pain in the anterior neck at the time of presentation. Pain was completely relieved in all patients at 17 days after starting the treatment. Sixty-four patients (68.8%) had fever at the time of presentation. At the end of therapy, it came to normal in all patients. At baseline, FT3 was 8.04 ± 3.17 pmol/L, FT4 was 27.82 ± 8.46 pmol/L, TSH was 1.01 (0.004-6.96) IU/mL, WBC was (11.68 ± 2.44) × 10^9^/L, neutrophils was (5.02 ± 1.92) × 10^9^/L and ESR was 68.88 ± 18.08 mm per hour at the time of presentation. FT3 (4.64 ± 1.09 pmol/L) and FT4 (13.82 ± 5.38 pmol/L) after the treatment were significantly lower than before the treatment (p < 0.01). TNF after the treatment was higher compared with before the treatment (p = 0.022). Compared with the value at the time of presentation, WBCs (6.59 ± 1.84) × 10^9^/L, neutrophils (3.12 ± 1.68) × 10^9^/L, ESR (14.04 ± 6.04 mL/hour) and CRP (4.10 ± 2.61 mg/L) were significantly lower after the treatment (p < 0.05 or p < 0.001).

**Table 1:** Clinical and biochemical characteristics of the subacute thyroiditis patients

Variables	Before treatment	After treatment	*p*-value
Female/Male (n/n)	65/28	65/28	—
Age (years)	46.8 ± 10.2	46.8 ± 10.2	—
Fever (%)	68.8%	0%	*p* < 0.001
Lobe pain (%)	100%	0%	*p* < 0.001
TSH (IU/mL) (0.34-5.44 IU/mL)	1.01 (0.004-6.96)	3.22 (0.12-5.94)	0.022
FT3 (pmol/L) (2.92-5.93 pmol/L)	8.04 ± 3.17	4.64 ± 1.09	*p* < 0.01
FT4 (pmol/L) (7.91-20.59 pmol/L)	27.82 ± 8.46	13.82 ± 5.38	*p* < 0.01
WBCs (10^9^/L) (4.0-10.0) × 10^9^/L	11.68 ± 2.44	6.59 ± 1.84	0.023
Neutrophils (10^9^/L) (1.80-6.30) × 10^9^/L	5.02 ± 1.92	3.12 ± 1.68	0.031
Mean ESR (0-20.00 mL/hour)	68.88 ± 18.08	14.04 ± 6.04	*p* < 0.001
CRP (0-5.00 mg/L)	33.01 ± 12.90	4.10 ± 2.61	*p* < 0.001

FT3 = free triiodothyronine; FT4 = free thyroxine; TSH = thyrotropin; WBC = white blood cell.

Data are presented as the mean ± SD or No. (percentage) of patients unless indicated otherwise. c. TSH levels and treatment duration are presented as the median and range (minimum-maximum).

### Symptoms relief and curative effect

**Figure 1:**
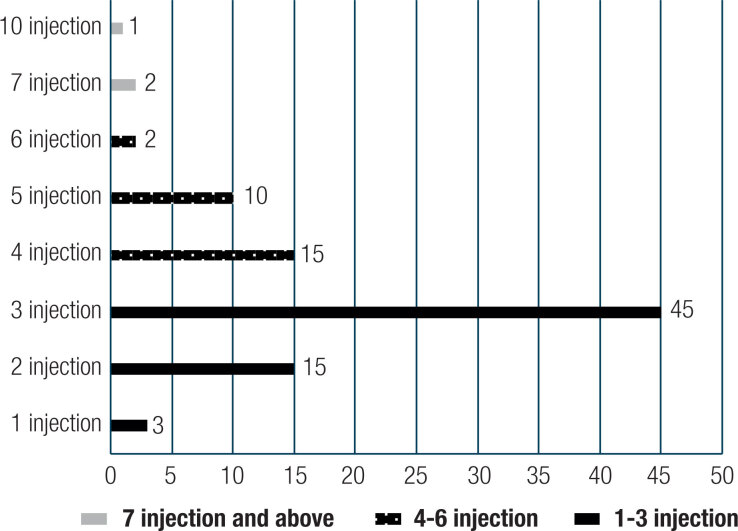
Distribution of the cumulative injection times from 1to 10 for the SAT patients.

None of the patients reported worsening of symptoms after starting the treatment. Thyroid pain instantly decreased, with scores below 3.0 following the administration of an injection. Approximately 92 out of the 93 patients, i.e., more than 99%, showed “recovery” results (VAS < 3 points) with no pain within two weeks. One patient (1%) showed an “ineffective” result (VAS > 4 points) with neck pain within two weeks, with up to ten injections, which took only 17 days altogether when the patient achieved a “recovery” therapeutic effect (VAS < 3 points). As shown in Figure 1, 63 patients (67.74%) avoided the relapse of thyroid pain within 3 injections, which occurred only within 3 days after the first injection. The pain in 27 (29.03%) patients disappeared completely after 4-6 injections. Only 3 patients (3.23%) were found to need more than 6 injections. The mean treatment cycle of the intrathyroidal injection was 3.98 days.

The changes in the patients’ pain scale scores during treatment are shown in Table 2. Thirty seconds after injection, 85 patients (91.4%) showed a perfect VAS score (VAS < 3). Seven (7.5%) patients showed a modest VAS score (4 < VAS < 7), and only one patients (1.1%) showed a poor VAS score (8 < VAS < 10). Sixty seconds after injection, 92 patients close to elimilate the pain (VAS < 3), only one patient had a poor effect (8 < VAS < 10). Three days after injection, 67.7% patients’ VAS score was lower than 3, 31.2% patients showed a modest VAS score (4 < VAS < 7). Only 1.1% patients’ pain was not relieved (8 < VAS < 10). As time go on, the percent of VAS < 3 increased gradually, and 4 < VAS < 7 decreased gradually. Seventeen days after injection, all patients showed a perfect effect (VAS < 3). Average patient satisfaction score was as high as 9.0.

**Table 2:** The evolution of the pain scale scores during treatment

Follow-up	Pain score of 0-3	Pain score of 4-7	Pain score of 8-10
30 seconds	85 (91.4)	7 (7.5)	1 (1.1)
60 seconds	92 (98.9)	0 (0)	1 (1.1)
3d	63 (67.7)	29 (31.2)	1 (1.1)
7d	82 (88.2)	11(11.8)	0 (0)
9d	90 (96.8)	3 (3.2)	0 (0)
16d	92 (98.9)	1 (1.1)	0 (0)
17d	93 (100)	0 (0)	0 (0)

#### Adverse reactions and relapse

Adverse reactions from the intrathyroidal injections were mild, and none of the patients were removed from the study because of side effects. Most of the patients complained of mild pain and swelling at the injection site, and these sensations usually lasted only for a few minutes. No cases of severe bleeding, such as hematoma, infection at the injection site or anaphylactic reactions, were observed after the injection. There were no reports of impaired blood sugar levels in patients following triamcinolone injection.

Surprisingly, there were no reports of relapse following the intrathyroidal injection of GCs using an insulin pen.

## DISCUSSION

In this study, we evaluated the efficacy and safety of ultrasound-guided intrathyroidal injection in the treatment of SAT. The simple injection could reduce local inflammation and easily improve symptoms via an insulin pen device. We devised a new way to ameliorate the symptoms, allow the disease to run its spontaneous course in an asymptomatic manner and avoid the systemic side effects of corticosteroids even using them. The results of this study indicate that our treatment is effective and safe.

Over 95% of people with SAT often present with moderate-to-severe pain (13). Once pain is observed in SAT patients, immediate treatment is required. There is no definitive therapy for painful SAT. Anti-inflammatory and pain relief therapies are considered the most effective treatments for SAT. Therefore, subacute thyroiditis has been treated with either nonsteroidal anti-inflammatory drugs (NSAIDs) or corticosteroids for years. The response to steroids is often more dramatic and quicker than the response to NSAIDs, but steroids are well known for their side effects over NSAIDs. As subacute thyroiditis is a self-limiting condition, a high dose of steroids might be irrelevant to some people. The basis for this dose has not yet been established by prospective studies. Prednisolone (PSL) 40 mg taken daily for 1-2 weeks followed by a gradual taper over 2-4 weeks or longer is the most popular recommendation. An initial PSL dosage of 20-40 mg daily is recommended for Asian SAT patients (7,8). A recent observational study showed that the treatment protocol of PSL 15 mg taken daily as the initial dosage with tapering by 5 mg every 2 weeks was effective and safe for Japanese patients (14). However, 20.5% of patients with SAT still require more than 8 weeks to recover from inflammation. Therefore, the treatment duration is at least 4-6 weeks and even longer at low doses.

Oral corticosteroids are important in the treatment of subacute thyroiditis. However, numerous adverse effects have been attributed to oral corticosteroids. Several retrospective reports have shown that long-term use of corticosteroids orally, even in low doses, is a significant independent predictor of adverse effects, such as increased appetite and weight, exacerbated insomnia, osteoporosis, and abnormal glucose regulation (15–17). The adverse effects of corticosteroids orally may lead to stop taking medicine during the treatment.

Fortunately, in our study, the patients immediately achieved neck pain relief through local injections. Compared with oral administration (systemic administration), local administration (percutaneous intrathyroid injection) has a direct effect on the gland, which can increase local drug concentrations rapidly, inhibit local inflammatory responses effectively, reduce the size of pathological tissues in the thyroid gland, and promote the recovery of the structure and function of thyroid follicles. Furthermore, compared with oral corticosteroid therapy, ultrasound-guided percutaneous corticosteroid injection for subacute thyroiditis is relatively safe and has less impact on patients’ body shape as well as glucose and lipid metabolism. The long-term recurrence rate during follow-up was significantly low.

Locally administered corticosteroids are already used as a common therapy in many hand and wrist disorders, representing a low risk to patients (18). Intrathyroidal corticosteroid injections were first reported in 1974 (19). The intrathyroidal administration of GCs with conventional syringes can reduce thyromegaly and the resulting symptoms in chronic thyroiditis, subacute thyroiditis and Graves’ disease promptly and sufficiently without side effects even after prolonged administration. In recent years, by referring to the intrathyroidal injection of immunosuppressants or GCs for the treatment of autoimmune thyroiditis, some scholars have treated SAT with the intrathyroidal injection of GCs and completed the whole process of puncture and drug injection with conventional syringes under the guidance and monitoring of ultrasound (20,21). However, the majority of relevant literature has small sample sizes and inconsistent conclusions, which limits their value for clinical reference. In a recent small sample study comparing the efficiency of intrathyroid steroid injection with conventional syringes to oral steroid intake in 32 patients with subacute thyroiditis, intrathyroid steroid injection was faster, safer and generally better tolerated by patients (22).

Surprisingly, different from previous study, we have some innovations and improvements. The insulin pen device relieves pain perception of injection because the device uses tiny needles and improves the accuracy of the injection (23). We have also used them for intrathyroidal injection and they exhibit the same advantages as those identified in diabetes treatment.

The injection drugs in the present study are fast-acting and effective. The mixture of lidocaine and triamcinolone was critical for this effect. Locally administered the mixture of corticosteroids and lidocaine are more effective than lidocaine or corticosteroids alone for pain control (24). These two drugs exhibit synergy in prolonging the duration of pain control effects. Lidocaine acts immediately, and triamcinolone exhibits sustained action (24,25). In our previous study (26), we discovered that lidocaine could inhibit the secretive function of thyroid follicular epithelial cells and avoid adenovirus-induced thyroid follicular epithelial cell apoptosis by inhibiting the pyroptosis pathway and the expression of inflammatory factors. All concentrations, including low, middle, and high levels of lidocaine, dramatically reduced the protein expression of IL-1α, IL-6, TNF-α, ELAVL1, NLRP3, caspase-1, and IL-1β in adenovirus-infected thyroid follicular epithelial cells, which indicated that the pyroptosis pathway was inhibited (26). In addition, lidocaine also decreased the relative levels of TG, TPO, T3, and T4 in adenovirus-infected thyroid follicular epithelial cells.

In our present study, the administration of lidocaine and triamcinolone via an insulin pen device has many advantages over conventional oral therapy or injection with conventional syringes. First, this method was as simple and easy to perform as an insulin pen injection, with the microneedle inserted into the edge of the pathological tissue stably and precisely under ultrasound guidance. The injection itself was almost painless and less likely to cause damage. However, the operation should be handled with care to avoid small hematomas. Complications can be avoided by improving the technique. Second, the accurate microdosing of lidocaine and triamcinolone by local injection had no obvious side effects compared to systemic administration. Third, the new technique shortened the duration of treatment and relieved pain immediately, rapidly and effectively in the appropriate clinical setting compared to conventional oral therapy.

In conclusion, we provide an innovative approach to the treatment of SAT. Ultrasound-guided intrathyroidal injection of glucocorticoids and lidocaine with an insulin pen is an effective and safe therapy. Injection is an ideal method of administration with respect to its rapid delivery, short duration, microdosing capacity, lack of obvious side effects, painlessness, ease of operation, and safety in patients with SAT. In future, we should designed a controlled trial compared with oral therapy.
